# Estimating influenza incidence using search query deceptiveness and generalized ridge regression

**DOI:** 10.1371/journal.pcbi.1007165

**Published:** 2019-10-01

**Authors:** Reid Priedhorsky, Ashlynn R. Daughton, Martha Barnard, Fiona O’Connell, Dave Osthus

**Affiliations:** 1 Los Alamos National Laboratory, Los Alamos, New Mexico, United States of America; 2 University of Colorado Boulder, Boulder, Colorado, United States of America; 3 Minnetonka Public Schools, Minnetonka, Minnesota, United States of America; Northeastern University, United States

## Abstract

Seasonal influenza is a sometimes surprisingly impactful disease, causing thousands of deaths per year along with much additional morbidity. Timely knowledge of the outbreak state is valuable for managing an effective response. The current state of the art is to gather this knowledge using in-person patient contact. While accurate, this is time-consuming and expensive. This has motivated inquiry into new approaches using internet activity traces, based on the theory that lay observations of health status lead to informative features in internet data. These approaches risk being deceived by activity traces having a coincidental, rather than informative, relationship to disease incidence; to our knowledge, this risk has not yet been quantitatively explored. We evaluated both simulated and real activity traces of varying deceptiveness for influenza incidence estimation using linear regression. We found that deceptiveness knowledge does reduce error in such estimates, that it may help automatically-selected features perform as well or better than features that require human curation, and that a semantic distance measure derived from the Wikipedia article category tree serves as a useful proxy for deceptiveness. This suggests that disease incidence estimation models should incorporate not only data about how internet features map to incidence but also additional data to estimate feature deceptiveness. By doing so, we may gain one more step along the path to accurate, reliable disease incidence estimation using internet data. This capability would improve public health by decreasing the cost and increasing the timeliness of such estimates.

## Author summary

While often considered a minor infection, seasonal flu kills many thousands of people every year and sickens millions more. The more accurate and up-to-date public health officials’ view of what the seasonal outbreak is, the more effectively the outbreak can be addressed. Currently, this knowledge is based on collating information on patients who enter the health care system. This approach is accurate, but it’s also expensive and slow. Researchers hope that new approaches based on examining what people do and share on the internet may work more cheaply and quickly. Some internet activity, however, has a history of correspondence with disease activity, but this relationship is coincidental rather than informative. For example, some prior work has found a correspondence between zombie-related social media messages and the flu season, so one could plausibly build accurate flu estimates using such messages that are then fooled by the appearance of a new zombie movie. We tested flu estimation models that incorporate information about this risk of deception, finding that knowledge of deceptiveness does indeed produce more accurate estimates; we also identified a method to estimate deceptiveness. Our results suggest that estimation models used in practice should use information about both how inputs maps to disease activity and also what the potential of each input to be deceptive is. This may get us one step closer to accurate, reliable disease estimates based on internet data, which would improve public health by making those estimates faster and cheaper.

## Introduction

Effective response to disease outbreaks depends on reliable estimates of their status. This process of identifying new outbreaks and monitoring ongoing ones—*disease surveillance*—is a critical tool for policy makers and public health professionals [1].

The traditional practice of disease surveillance is based upon gathering information from in-person patient visits. Clinicians make a diagnosis and report that diagnosis to the local health department. These health departments aggregate the reports to produce local assessments and also pass information further up the government hierarchy to the national health ministry, which produces national assessments [2]. Our previous work describes this process with a mathematical model [3].

This approach is accepted as sufficient for decision-making [4, 5] but is expensive, and results lag real time by anywhere from a week [6] to several months [7, 8]. Novel surveillance systems that use disease-related internet activity traces such as social media posts, web page views, and search queries are attractive because they would be faster and cheaper [9, 10]. One can conjecture that an increase of influenza-related web searches is due to an increase in flu observations by the public, which in turn corresponds to an increase in real influenza activity. These systems use statistical techniques to estimate a mapping from past activity to past traditional surveillance data, then apply that map to current activity to predict current (but not yet available) traditional surveillance data, a process known as *nowcasting*.

One specific concern with this approach is that these models can learn coincidental, rather than informative, relationships [3]. For example, Bodnar and Salathé found a correlation between zombie-related Twitter messages and influenza [11]; one could plausibly use these data to build an accurate flu model that would be fooled by the appearance of a new zombie movie.

**Fig 1:**
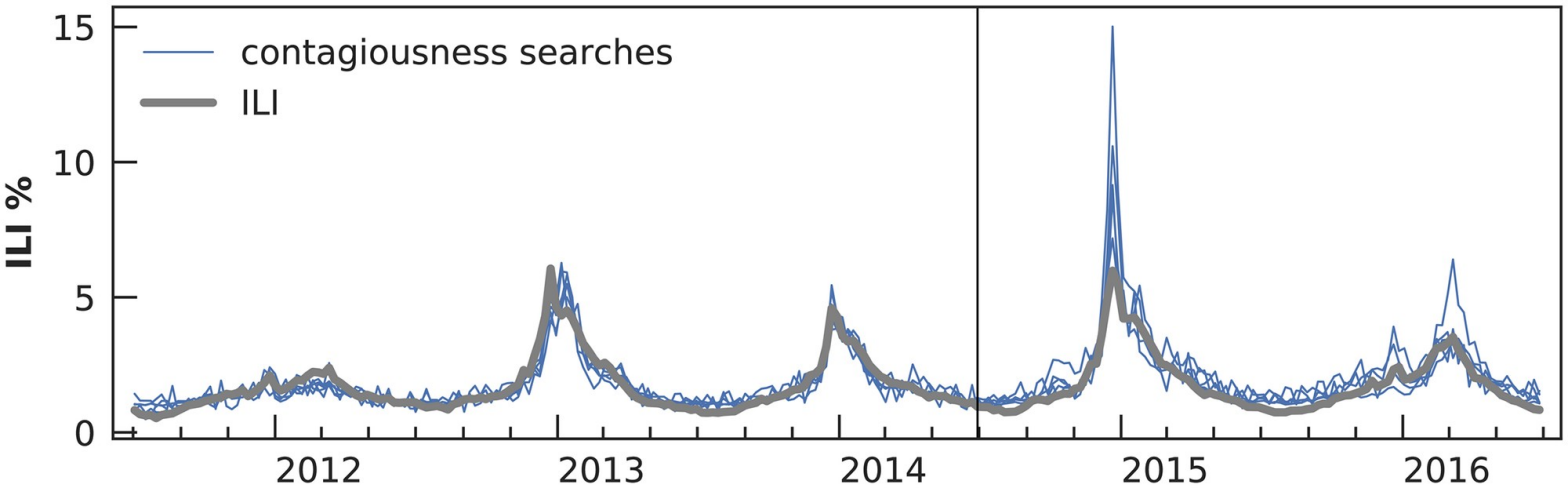
Simple deceptive models for influenza-like illness (ILI). This figure shows five one-feature models that map U.S. Google volume for contagiousness-related searches to ILI data from the CDC (“how long are you contagious”, “flu contagious”, “how long am i contagious”, “influenza contagious”, “when are you contagious”). We fit using ordinary least squares linear regression over the first three seasons of the study period. Despite a good fit during the training period, these models severely overestimate the peaks of the fourth and fifth seasons, demonstrating that a model’s history of accuracy does not yield accurate predictions in the future if it uses deceptive input features, such as these contagiousness searches. Our *deceptiveness* metric quantifies the risk of such divergence.

More quantitatively, Ginsberg et al. built a flu model using search queries selected from 50 million candidates by a purely data-driven process that considered correlation with influenza-like-illness in nine regions of the United States [12]. Of the 45 queries selected by the algorithm for inclusion in the model, 6 (13%) were only weakly related to influenza (3 categorized as “antibiotic medication”, 2 “symptoms of an unrelated disease”, 1 “related disease”). Of the 55 next-highest-scoring candidates, 8 (15%) were weakly related (3, 2, and 3 respectively) and 19 (35%) were “unrelated to influenza”, e.g. “high school basketball”. That is, even using a high-quality, very computationally expensive approach that leveraged demonstrated historical correlation in nine separate geographic settings, one-third of the top 100 features were weakly related or not related to the disease in question. Fig 1 illustrates this problem for flu using contagiousness-related web searches.

Such features, with a dubious real link to the quantity of interest, pose a risk that the model may perform well during training but then provide erroneous estimates later when coincidental relationships fail, especially if they do so suddenly. We have previously proposed a metric called *deceptiveness* to quantify this risk [3]. This metric quantifies the fraction of an estimate that depends on noise rather than signal and is a real number between 0 and 1 inclusive. Deceptiveness of zero indicates the estimate is based completely on informative relationships (signal), deceptiveness of one indicates the estimate is based completely on coincidental relationships (noise), and deceptiveness of $\frac{1}{2}$ indicates an equal mix of the two. It is important to note that deceptiveness has nothing to do with semantic relatedness and its true values are unknowable; however, below we show that it can be usefully estimated using relatedness information. We hypothesize that disease nowcasting models that leverage the deceptiveness of input features (perhaps estimated) have better accuracy than those that do not.

This is an important question because disease forecasting is improved by better nowcasting. For example, Brooks et al.’s top-performing entry [13] to the CDC’s flu forecasting challenge [14] was improved by nowcasting. Lu et al. tested autoregressive nowcasts using several internet data sources and found that they improved 1-week-ahead forecasts [15]. Kandula et al. measured the benefit of nowcasting to their flu forecasting model at 8–35% [16]. Finally, our own work shows that a Bayesian seasonal flu forecasting model using ordinary differential equations benefits from filling a one-week reporting delay with internet-based nowcasts [17].

The present work tests this hypothesis using five seasons of influenza-like-illness (ILI) in the United States (2011–2016). We selected U.S. influenza because high-quality reference data are easily available and because it is a pathogen of great interest to the public health community. Although flu is often considered a mild infection, it can be quite dangerous for some populations, including older adults, children, and people with underlying health conditions. Typical U.S. flu seasons kill ten to fifty thousand people annually [5].

Our experiment is a simulation study followed by validation using real data. This lets us test our approach using fully-known deceptiveness as well as a more realistic setting with estimated deceptiveness. We trained linear estimation models to nowcast ILI using an extension of ridge regression [18] called *generalized ridge* [19] or *gridge regression* that lets us apply individual weights to each feature, thus expressing a prior belief on their value: higher value for lower deceptiveness. We used three classes of input features: (1) synthetic features constructed by adding plausible noise patterns to ILI, (2) Google search volume on query strings related to influenza, and (3) Google search volume on inferred topics related to influenza.

We found that accurate deceptiveness knowledge did indeed reduce prediction error, and in the case of the automatically-generated query string features, as much or more than topic features that require human curation. We also found that semantic distance as derived from the Wikipedia article category tree served as a useful proxy for deceptiveness.

The remainder of this paper is organized as follows. We next describe our data sources, regression approach, and experiment structure. After that, we describe our results and close with their implications and suggestions for future work.

## Methods

Our study period was five consecutive flu seasons, 2011–2012 through 2015–2016, using weekly data. The first week in the study was the week starting Sunday, July 3, 2011, and the last week started Sunday, June 26, 2016, for a total of 261 consecutive weeks. We considered each season to start on the first Sunday in July, and the previous season to end on the day before (Saturday).

We used gridge regression to fit input features to U.S. ILI over a subset of the first three seasons, then used the fitted coefficients to estimate ILI in the fourth and fifth seasons. (We used this training schedule, rather than training a new model for each week as one might do operationally, in order to provide a more challenging setting to better differentiate the models). We assessed accuracy by comparing the estimates to ILI using three metrics: *r*^2^ (the square of Pearson correlation), root mean squared error (RMSE), and hit rate.

The experiment is a full factorial experiment with four factors, yielding a total of 225 models:

1. **Class of input features** (3 levels): synthetic features, search query string volume, and search topic volume.
2. **Training period** (3): one, two, or three consecutive seasons.
3. **Noise added to deceptiveness** (5): perfect knowledge of deceptiveness to none at all.
4. **Model type** (5): ridge regression and four levels of gridge regression.

This procedure is implemented in a Python program and Jupyter notebook, which are available and documented on GitHub at https://github.com/reidpr/quac/tree/master/experiments/2019_PLOS-Comp-Bio_Deceptiveness.

The remainder of this section describes our data sources, regression algorithm, experimental factors, and assessment metrics in detail.

### Data sources

We used four types of data in this experiment:

1. **Reference data.** U.S. national ILI from the Centers for Disease Control and Prevention (CDC). This is a weekly estimate of influenza incidence.
2. **Synthetic features.** Weekly time series computed by adding specific types of systematic and random noise to ILI. These simulated features have known deceptiveness.
3. **Flu-related concepts.** We used the crowdsourced Wikipedia category tree to enumerate a set of concepts and estimate the semantic relatedness of each to influenza.
4. **Real features.** Two types of weekly time series: Google search query strings and Google search topics. These features are based on the flu-related concepts above and use estimated flu relatedness as a proxy for deceptiveness.

This section explains the format and quirks of the data, how we obtained them, and how readers can also obtain them.

#### Reference data: U.S. influenza-like illness (ILI) from CDC

*Influenza-like illness* (ILI) is a syndromic metric that estimates influenza *incidence*, i.e., the number of new flu infections. It is the fraction of patients presenting to the health care system who have symptoms consistent with flu and no alternate explanation [6]. The basic process is that certain clinics called *sentinel providers* report the total number of patients seen during each interval along with those diagnosed with ILI. Local, provincial, and national governments then collate these data to produce corresponding ILI reports [6].

U.S. ILI values tend to range between 1–2% during the summer and 7–8% during a severe flu season [6, 20]. While an imperfect measure (for example, it is subject to reporting and behavior biases if some groups, like children, are more commonly seen for ILI [21]), it is considered sufficiently accurate for decision-making purposes by the public health community [4, 5].

In this study, we used weekly U.S. national ILI downloaded from the Centers for Disease Control and Prevention (CDC)’s FluView website [22] on December 21, 2016, six months after the end of the study period. This delay is enough for reporting backfill to settle sufficiently [17]. Fig 1 illustrates these data, and they are available as an Excel spreadsheet in S1 Dataset.

#### Synthetic features: Computed by us

These simulated features are intended to model a plausible way in which internet activity traces with varying usefulness might arise. Their purpose is to provide an experimental setting with features that are sufficiently realistic and have known deceptiveness.

Each synthetic feature *x* is a random linear combination of ILI *y*, Gaussian random noise *ε*, and systematic noise *σ* (all vectors with 261 elements, one for each week):

$$\begin{array}{c} x=w_{\text{i}}y+w_{\text{r}}\varepsilon +w_{\text{s}}\sigma \end{array}$$ (1)

$$\begin{array}{c} 1=w_{\text{i}}+w_{\text{r}}+w_{\text{s}} \end{array}$$ (2)

Because a feature’s deceptiveness (*g* ∈ [0, 1]) is the fraction of the signal attributable to noise, here we use simply the weight of its systematic noise: *g* = *w*_s_. Importantly, these features are synthetic, so we know their true deceptiveness and can assess its impact. In the real world, however, deceptiveness is unknowable and must be estimated using proxies, as we discuss in the next section.

Random noise *ε* is a random multivariate normal vector with standard deviation 1. Systematic noise *σ* is a random linear combination of seven basis functions *σ*_*j*_:

$$\begin{array}{c} w_{\text{s}}\sigma =\sum_{j=1}^{7}w_{\text{s}j}\sigma_{j} \end{array}$$ (3)

$$\begin{array}{c} w_{\text{s}}=\sum_{j=1}^{7}w_{\text{s}j} \end{array}$$ (4)

**Fig 2:**
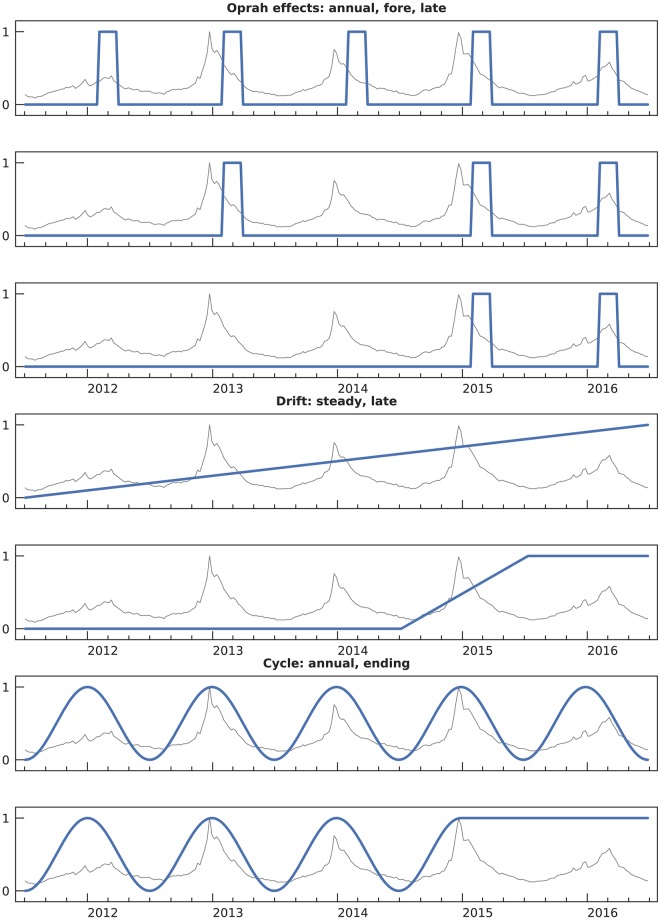
Systematic noise basis functions for synthetic features. These functions model plausible mechanisms of how features are affected by exogenous events.

These basis functions, illustrated in Fig 2, simulate sources of systematic noise for internet activity traces. They fall into three classes:

- **Oprah effect [3]:** 3 types. These simulate pulses of short-lived traffic driven by media interest. For example, U.S. Google searches for measles were 10 times higher in early 2015 than any other time during the past five years [23], but measles incidence peaked in 2014 [24].
  The three specific bases are: *annual* *σ*_1_, a pulse every year shortly after the flu season peak; *fore* *σ*_2_, pulses during both the training and test seasons (second, fourth, and fifth); and *late* *σ*_3_, pulses only during the test seasons (fourth and fifth). The last creates features with novel divergence after training is complete, producing deceptive features that cannot be detected by correlation with reference data.
- **Drift:** 2 types. These simulate steadily changing public interest. For example, as the case definition of autism was modified, the number of individuals diagnosed with autism increased [25].
  The two bases are: *steady* *σ*_4_, a slow change over the entire study period of five seasons, and *late* *σ*_5_, a transition from one steady state to another over the fourth season. The latter again models novel divergence.
- **Cycle:** 2 types. This simulates phenomena that have an annual ebb and flow correlating with the flu season. An example is the U.S. basketball season noted above.
  The two bases are: *annual* *σ*_6_, cycles continuing for all five seasons, and *ending* *σ*_7_, cycles that end after the training seasons. The latter again models novel divergence.

In order to build one feature, we need to sample the nine elements of the weight vector *w* = (*w*_i_, *w*_r_, *w*_s1_, …*w*_s7_), which sums to 1. This three-step procedure is as follows. First, *w*_i_, *w*_r_, and *w*_s_ are sampled from a Dirichlet distribution:

$$\begin{array}{c} \left(w_{\text{i}},w_{\text{r}},w_{\text{s}}\right)∼\text{Dir}\left(1.0,0.5,1.5\right) \end{array}$$ (5)

$$\begin{array}{c} \text{E}\left(w_{\text{i}}\right)=\frac{1}{3},\;\text{E}\left(w_{\text{r}}\right)=\frac{1}{6},\;\text{E}\left(w_{\text{s}}\right)=\frac{1}{2} \end{array}$$ (6)

Next, the relative weight of the three types of systematic noise is sampled:

$$\begin{array}{c} \left(w_{\text{so}},w_{\text{sd}},w_{\text{sc}}\right)∼w_{\text{s}}\text{Dir}\left(0.3,0.3,0.3\right) \end{array}$$ (7)

Finally, all the weight for each type is randomly assigned with equal probability to a single basis function:

$$\begin{array}{c} \left(w_{\text{s}1},w_{\text{s}2},w_{\text{s}3}\right)∼w_{\text{so}}\text{Multinomial}\left(1,\left[\frac{1}{3},\frac{1}{3},\frac{1}{3}\right]\right) \end{array}$$ (8)

$$\begin{array}{c} \left(w_{\text{s}4},w_{\text{s}5}\right)∼w_{\text{sd}}\text{Multinomial}\left(1,\left[\frac{1}{2},\frac{1}{2}\right]\right) \end{array}$$ (9)

$$\begin{array}{c} \left(w_{\text{s}6},w_{\text{s}7}\right)∼w_{\text{sc}}\text{Multinomial}\left(1,\left[\frac{1}{2},\frac{1}{2}\right]\right) \end{array}$$ (10)

**Fig 3:**
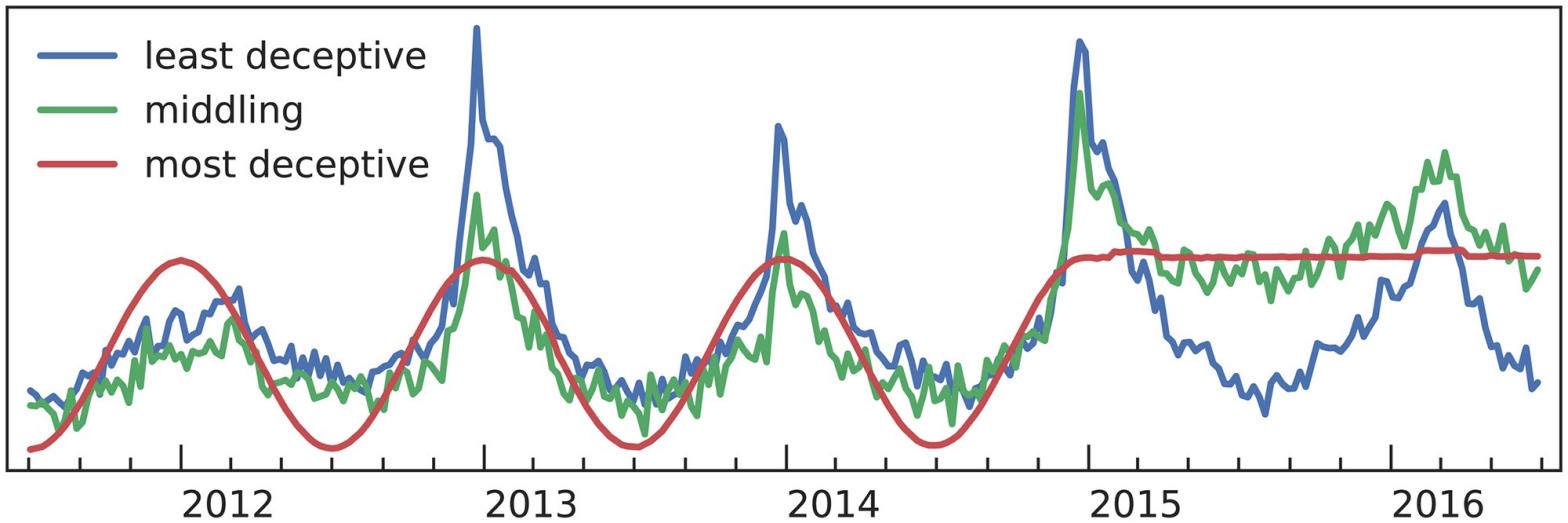
Example synthetic features. This figure shows the least and most deceptive features and a third with medium deceptiveness.

**Fig 4:**
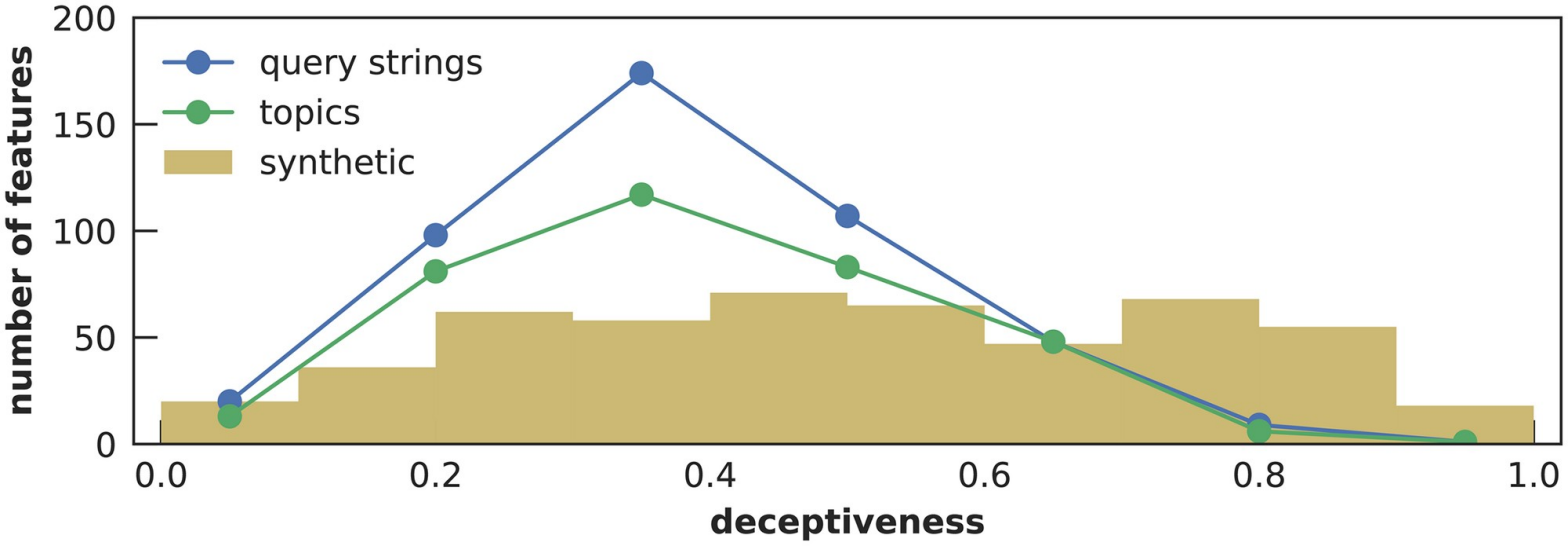
Deceptiveness histogram for all three feature types. Synthetic features have continuous deceptiveness and are grouped into ten bins, while the two types of search features have seven discrete deceptiveness levels.

This procedure yields a variety of high- and low-quality synthetic features. We sampled 500 of them. Fig 3 shows three examples, and Fig 4 shows the deceptiveness histogram. The features we used are available in S3 Dataset.

#### Flu-related concepts: Wikipedia

In order to build a flu nowcasting model based on web search queries that incorporates an estimate of deceptiveness, we first need a set of influenza-related concepts. We used the Wikipedia inter-article link graph to generate a set of candidate concepts and the Wikipedia article category hierarchy to estimate the semantic relatedness of each concept to influenza, which we use as a proxy for deceptiveness. An important advantage of this approach is that it is automated and easily generalizable to other diseases.

Wikipedia is a popular web-based encyclopedia whose article content and metadata are crowdsourced [26]. We used two types of metadata from a dataset [27] collected March 24, 2016 for our previous work [9] using the Wikipedia API.

First, Wikipedia articles contain many hyperlinks to other articles within the encyclopedia. This work used the article “Influenza” and the 572 others it links to, including clearly related articles such as “Infectious disease” and apparently unrelated ones such as “George W. Bush”, who was the U.S. president immediately prior to 2009 H1N1.

Second, Wikipedia articles are leaves in a category hierarchy. Both articles and categories have one or more parent categories. For example, one path from “Influenza” to the top of the tree is: *Healthcare-associated infections*, *Infectious diseases*, *Diseases and disorders*, *Health*, *Main topic classifications*, *Articles*, and finally *Contents*. This tree can be used to estimate semantic relatedness between two articles. The number of levels one must climb the tree before finding a common category is a metric called *category distance* [9]; the distance between an article and itself is 1. For example, the immediate categories of “Infection” include *Infectious diseases*. Thus, the distance between these two articles is 2, because we had to ascend two levels from “Influenza” before discovering the common category *Infectious diseases*.

We used category distance between each of the 573 articles and “Influenza” to estimate the semantic relatedness to influenza. The minimum category distance was 1 and the maximum 7.

The basic intuition for this approach is that Wikipedia category distance is a reasonable proxy for how related a concept is to influenza, and this relatedness is in turn a reasonable proxy for deceptiveness. For example, consider a distance-1 feature and a distance-7 feature that are both highly correlated with ILI. Standard linear regression will give equal weight to both features. However, we conjecture that the distance-7 feature’s correlation is more likely to be spurious than the distance-1’s; i.e., we posit that the distance-7 feature is more deceptive. Thus, we give the distance-1 feature more weight in the regression, as described below.

Because category distance is a discrete variable d∈[1,7]∩ℤ, while deceptiveness *g* ∈ [0, 1] is continuous, we convert category distance into a deceptiveness estimate $\hat{g}$ as follows:

$$\begin{array}{c} \hat{g}=\left(1-2\epsilon \right)\frac{d-1}{6}+\epsilon \end{array}$$ (11)

The purpose of *ϵ* ≠ 0 is to ensure that features with minimum category distance of 1 receive regularization from the linear regression, as described below. In our initial data exploration, the value of *ϵ* had little effect, so we used *ϵ* = 0.05; therefore, $\hat{g}\in \{0.05,0.20,0.35,0.50,0.65,0.80,0.95\}$. We emphasize that category distance is already a noisy proxy for deceptiveness. Even with zero noise added to deceptiveness, $\hat{g}\neq g$.

It is important to realize that because Wikipedia is continually edited, metadata such as links and categories change over time. Generally, mature topic areas such as influenza and infectious disease are more stable than, for example, current events. The present study assumes that the dataset we used is sufficiently correct despite its age; i.e., freshly collected links and categories might be somewhat different but not necessarily more correct.

The articles we used and their category distances are in S2 Dataset.

#### Real features: Google searches

Typically, each feature for internet-based disease surveillance estimates public interest in a specific concept. This study uses Google search volume as a measure of public interest. By mapping our Wikipedia-derived concepts to Google search queries, we obtained a set of queries with estimated deceptiveness. Then, search volume over time for each of these queries, as well as their deceptiveness, are input for our algorithms.

We tested two types of Google searches. *Search query strings* are the raw strings typed into the Google search box. We designed an automated procedure to generate query strings from Wikipedia article titles. *Search topics* are concepts assigned to searches by Google using proprietary and unspecified algorithms. We built a map from Wikipedia article titles to topics manually.

Our procedure to map articles to query strings is:

1. Decode the percent notation in the title portion of the article’s URL [28].
2. Change underscores to spaces.
3. Remove parentheticals.
4. Approximate non-ASCII characters with ASCII using the Python unidecode package [29].
5. Change upper case letters to lower case. (This serves a simplifying rather than necessary purpose, as the Google Trends API is case-insensitive.)
6. Remove all characters other than alphanumeric, slash, single quote, space, and hyphen.
7. Remove *stop phrases* we felt were unlikely to be typed into a search box. Matches for the following regular expressions were removed (note leading and trailing spaces):
  - “ and\b”
  - “^global ”
  - “^influenza .? virus subtype ”
  - “^list of ”
  - “^the ”

This produces a query string for all 573 articles. The map is 1-to-1: each article maps to exactly one query string, and each query string maps to exactly one article. The process is entirely automated once the list of stop phrases is developed.

Google search topics is a somewhat more amorphous concept. Searches are assigned to topics by Google’s proprietary machine learning algorithms, which are not publically available [30]. A given topic is identified by its name or a hexadecimal code. For example, the query string “apple” might be assigned to “Apple (fruit)” or “Apple (technology company)” based on the content of the full search session or other factors.

To manually build a mapping between Wikipedia articles and Google search topics, we entered the article title and some variations into the search box on the Google Trends website [23] and then selected the most reasonable topic named in the site’s auto-complete box. The topic code was in the URL. If the appropriate topic was unclear, we discussed it among the team. Not all articles had a matching topic; we identified 363 topics for the 573 articles (63%). Among these 363 articles, the map is 1-to-1.

Table 1 shows a few examples of both mappings, and Fig 4 shows the deceptiveness histograms.

**Table 1:** Sample Wikipedia articles and the queries to which they map.

Article	Query string	Topic name	Topic code
Sense (molecular biology)	sense	Sense (Molecular biology)	/m/0dpw95
Influenza A virus subtype H9N2	h9n2	Influenza A virus subtype H9N2 (Virus)	/m/0b3dc1
George W. Bush	george w bush	George W. Bush (43rd U.S. President)	/m/09b6zr
Löffler’s syndrome	loffler’s syndrome	*none*	*none*

We downloaded search volume for both query strings and topics from the Google Health Trends API [31] on July 31, 2017. This gave us a weekly time series for the United States for each search query string and topic described above. These data appear to be a random sample, changing slightly from download to download.

**Fig 5:**
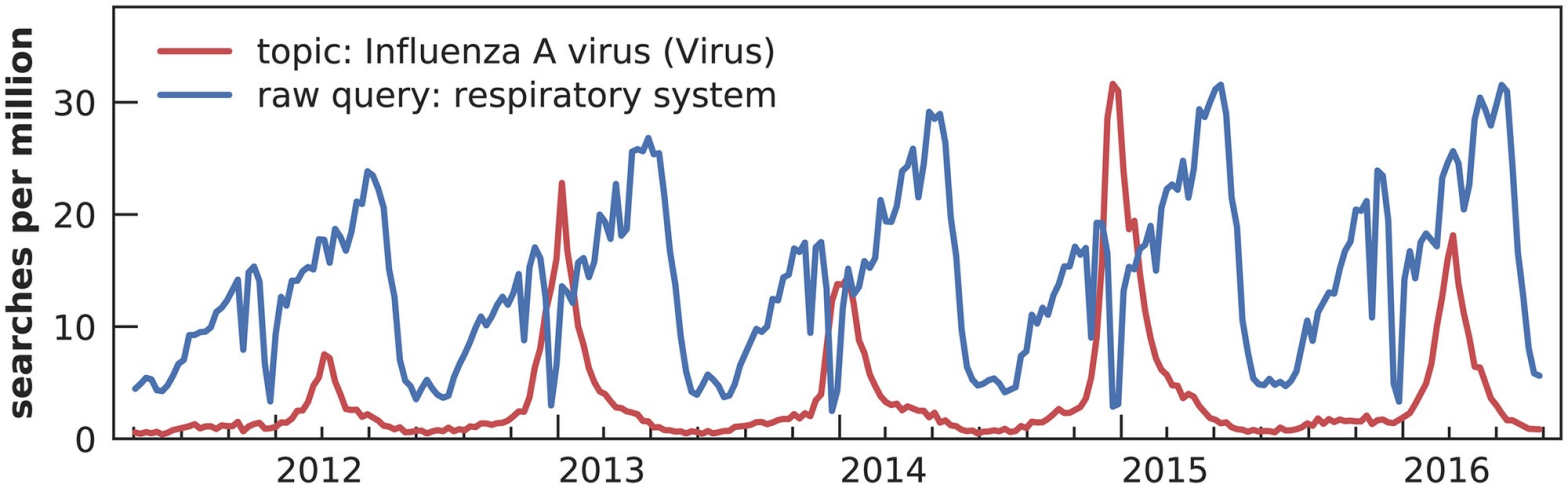
Search volume for two queries presented to our model. The topic “Influenza A virus (Virus)” shows a seasonal pattern roughly corresponding to ILI, while the raw query “respiratory system” shows a seasonal pattern that does not correspond to ILI.

Each element of the time series is the probability that the reported search session contains the string or topic, multiplied by 10 million. Searches without enough volume to exceed an unspecified privacy threshold are set to zero, i.e., we cannot distinguish between few searches and no searches. For this reason, we removed from analysis searches with more than 2/3 of the 261 weeks having a value of zero. This resulted in 457 usable of 573 query strings (80%) and 349 of 363 topics (96%). Fig 5 shows two of these time series.

Our map and category distances are in S2 Dataset, and the search queries used in Fig 1 are in S4 Dataset. Google’s terms of service prohibit redistribution of the search volume data. However, others can request the data using the same procedure we used [32]; instructions accompany the experiment source code.

### Gridge regression

Linear regression is a popular approach for mapping features (inputs) to observations (output). This section describes the algorithm and the extensions we used in our experiment to incorporate deceptiveness information.

The model for linear regression is

$$\begin{array}{c} y=X\beta +\varepsilon \end{array}$$ (12)

where *y* is an *N* × 1 observation vector, *X* = [1, *x*_1_, *x*_2_, …, *x*_*p*_] is an *N* × (*p* + 1) feature matrix where *x*_*i*_ is the *N* × 1 standardized feature vector (i.e., mean centered and standard deviation scaled) corresponding to feature *i*, *β* is a (*p* + 1) × 1 coefficient vector, *ε* ∼ MVN(0, *σ*^2^ *I*), where MVN(*μ*, Σ) is a multivariate normal distribution with mean *μ* and covariance matrix Σ, *σ*^2^ > 0 is a scalar, and *I* is an *N* × *N* identity. Standardizing the features of *X* is a convention that places all features on the same scale.

The goal of linear regression is to find the estimate of *β* that minimizes the sum-of-squared residual errors. The *ordinary least squares* (OLS) estimator ${\hat{\beta }}^{\text{OLS}}$ solves the following:

$$\begin{array}{c} {\hat{\beta }}^{\text{OLS}}=\underset{\beta }{arg min}\sum_{j=1}^{N}{\left(y_{j}-\beta_{0}-\sum_{i=1}^{p}\beta_{i}x_{ji}\right)}^{2} \end{array}$$ (13)

or in matrix form:

$$\begin{array}{c} {\hat{\beta }}^{\text{OLS}}={\left(X^{\prime }X\right)}^{-1}Xy \end{array}$$ (14)

${\hat{\beta }}^{\text{OLS}}$ is the unbiased, minimum variance estimator of *β*, assuming *ε* is normally distributed [33, ch. 1–2]. A prediction corresponding to a new feature vector $\ddot{x}$ is ${\hat{y}}^{\text{OLS}}=\ddot{x}{\hat{\beta }}^{\text{OLS}}$.

While ${\hat{y}}^{\text{OLS}}$ is unbiased, it is often possible to construct an estimator with smaller *expected prediction error*—i.e., with predictions on average closer to the true value—by introducing some amount of bias through *regularization*, which is the process of introducing additional information beyond the data. Also, regularization can make regression work in situations with more features than observations, like ours.

One popular regularization method is called *ridge regression* [18], which extends OLS by encouraging the coefficients *β* to be small. This minimizes:

$$\begin{array}{c} {\hat{\beta }}_{\lambda }^{\text{ridge}}=\underset{\beta }{arg min}\{\sum_{j=1}^{N}{\left(y_{j}-\beta_{0}-\sum_{i=1}^{p}\beta_{i}x_{ji}\right)}^{\;\;2}+\lambda \sum_{i=1}^{p}\beta_{i}^{2}\} \end{array}$$ (15)

or equivalently:

$$\begin{array}{c} {\hat{\beta }}_{\lambda }^{\text{ridge}}={\left(X^{\prime }X+\lambda I\right)}^{-1}X^{\prime }y. \end{array}$$ (16)

The additional parameter λ ≥ 0 controls the strength of regularization. When λ = 0, this is equivalent to OLS. As λ increases, the coefficient vector *β* is constrained towards zero more vociferously. Ridge regression applies the same degree of regularization to each feature, as λ is common to all features.

A second extension, called *generalized ridge regression* [19] or *gridge regression*, adds a feature-specific modifier *κ*_*i*_ to the regularization:

$$\begin{array}{c} {\hat{\beta }}_{\lambda \kappa }^{\text{gridge}}=\underset{\beta }{arg min}\{\sum_{j=1}^{N}{\left(y_{j}-\beta_{0}-\sum_{i=1}^{p}\beta_{i}x_{ji}\right)}^{\;\;2}+\lambda \sum_{i=1}^{p}\kappa_{i}\beta_{i}^{2}\} \end{array}$$ (17)

*κ*_*i*_ ≥ 0 adjusts the regularization penalty individually for each feature (ridge regression is a special case where *κ*_*i*_ = 1 ∀ *i*). Gridge retains closed-form solvability:

$$\begin{array}{c} {\hat{\beta }}_{\lambda \kappa }^{\text{gridge}}={\left(X^{\prime }X+\lambda K\right)}^{-1}X^{\prime }y \end{array}$$ (18)

where *K* is a diagonal matrix with *κ*_*i*_ on the diagonal and zero on the off-diagonals.

Gridge regression allows us to incorporate feature-specific deceptiveness information by making *κ*_*i*_ a function of feature *i*’s deceptiveness. The more deceptive feature *i*, the larger *κ*_*i*_.

### Experiment factors

Our experiment had 225 conditions. This section describes its factors: input feature class (3 levels), training period (3), deceptiveness noise added (5), and regression type (5).

#### Input feature class

We tested three classes of input features:

1. **Synthetic.** Randomly generated transformations of ILI, as described above.
2. **Search query string.** Volume of Google searches entered directly by users, as described above.
3. **Search topic.** Volume of Google search topics inferred by Google’s proprietary algorithms, as described above.

Each feature comprises a time series of weekly data, with frequency and alignment matching our ILI data.

#### Training period

We tested three different training periods: 1st through 3rd seasons inclusive (three season), 2nd and 3rd (two seasons), and 3rd only (one season). Because the 4th season contains transitions in the synthetic features, we did not use it for training even when testing on the 5th season.

#### Deceptiveness noise added

The primary goal of our study is to evaluate how much knowledge of feature deceptiveness helps disease incidence models. In the real world, this knowledge will be imperfect. Thus, one of our experiment factors is to vary the quality of feature deceptiveness knowledge.

Our basic approach is to add varying amounts of noise to the best available estimate of each feature’s deceptiveness $\hat{g}\in [0,1]$. Recall that for synthetic features, $\hat{g}=g$ is known exactly, while for the search-based features, $\hat{g}$ is an estimate based on the Wikipedia category distance.

To compute the noise-added deceptiveness ${\tilde{g}}_{i}$ for feature *i*, for noise added *γ*, we simply select a random other feature *j* and mix with its deceptiveness: ${\tilde{g}}_{i}=(1-\gamma ){\hat{g}}_{i}+\gamma {\hat{g}}_{j}$. There are five levels of this factor:

- Zero noise: *γ* = 0, i.e., the model gets the best available estimate of *g*_*i*_.
- Low noise: *γ* = 0.05.
- Medium noise: *γ* = 0.15.
- High noise: *γ* = 0.4.
- Total noise: *γ* = 1, i.e., the model gets no correct information at all about *g*_*i*_.

Models do not know what condition they are in; they get only ${\tilde{g}}_{i}$, not *γ*.

#### Regression type

We tested five types of gridge regression:

1. Ridge regression: *κ*_*i*_ = 1, i.e., ignore deceptiveness information.
2. Threshold gridge regression: keep features with category distance *d*_*i*_ ≤ 3 and discard them otherwise, as in [9]. This is implemented as a threshold ${\tilde{g}}_{i}=0.35$, which is applicable to both search and synthetic features (which have no *d*_*i*_).
  $$\begin{array}{c} \kappa_{i}=\{\begin{array}{cc} 0.1 & \text{if}\;{\tilde{g}}_{i}\leq 0.35\\ 1 & \text{otherwise} \end{array} \end{array}$$ (19)
3. Linear fridge: $\kappa_{i}={\tilde{g}}_{i}$.
4. Quadratic fridge: $\kappa_{i}={\tilde{g}}_{i}^{2}$.
5. Quartic fridge: $\kappa_{i}={\tilde{g}}_{i}^{4}$.

These levels are in rough ascending order of deceptiveness importance. (We additionally tested, but do not report, a few straw-man models to help identify bugs in our code).

All models used λ = 150.9. This value was obtained by 10-fold cross-validation [34]. For each model, we tested 41 values of λ evenly log-spaced between 10^−1^ and 10^7^; each fold fitted a model on the 9 folds left in and then evaluated its RMSE on the one fold left out. The λ with the lowest mean RMSE (plus a bias of up to 0.02 to encourage λs in the middle of the range) across the 10 folds, was reported as the best λ for that model. We then used the mean of these best λs for our experiment.

### Assessment of models

To evaluate a model, we apply its coefficients learned during the training period to input features during the 52 weeks of the fourth and fifth seasons respectively, yielding estimated ILI $\hat{y}$. We then compare $\hat{y}$ to reference ILI *y* for each of the two test seasons. For each model and metric, this yields two scalars.

We report three metrics:

1. ***r*^2^**, the square of the Pearson correlation *r*. Most previous work reports this unitless metric.
2. **Root mean squared error** (RMSE), defined as:
  $$\begin{array}{c} \sqrt{\frac{1}{N}\sum_{j=1}^{N}{\left(y_{j}-{\hat{y}}_{j}\right)}^{2}} \end{array}$$ (20)
  has interpretable units of ILI.
3. **Hit rate** is a measure of how well a prediction captures the direction of change. It is defined as the fraction of weeks when the direction of the prediction (increase or decrease) matches the direction of the reference data [10]:
  $$\begin{array}{c} \frac{\sum_{j=2}^{N}\text{sign}\left(y_{j}-y_{j-1}\right)\overset{?}{=}\text{sign}\left({\hat{y}}_{j}-{\hat{y}}_{j-1}\right)}{N-1} \end{array}$$ (21)
  Because it captures the trend (is the flu going up or down?), it directly answers a simple, relevant, and practical public health question.

## Results

**Fig 6:**
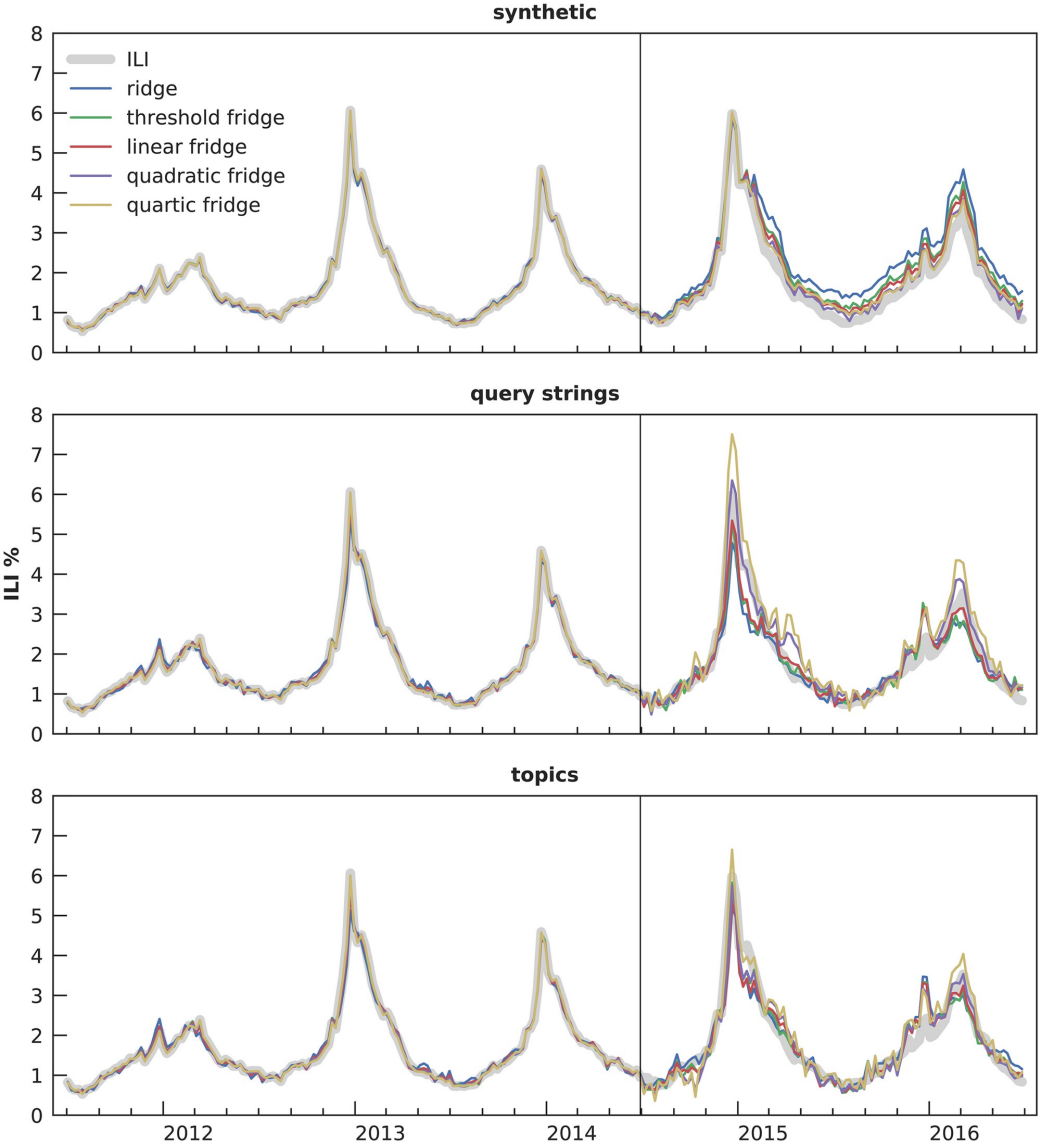
ILI predictions for zero-added-noise, 3-season-trained models. This figure shows the 15 models in the “ideal” situation: no noise added to deceptiveness, and trained on all three training seasons. The different types of gridge regression show subtle yet distinct differences, with the models taking into account deceptiveness more generally being closer to the ILI reference data.

Output of our regression models is illustrated in Fig 6, which shows 15 selected conditions. These conditions are close to what would be done in practice: use all available training information and add no noise. The differences between gridge regression types are subtle, but they are real, and close examination shows that the stronger gridge models that place higher importance on deceptiveness information are closer to the ILI reference data. The remainder of this section analyzes these differences across all the conditions.

**Fig 7:**
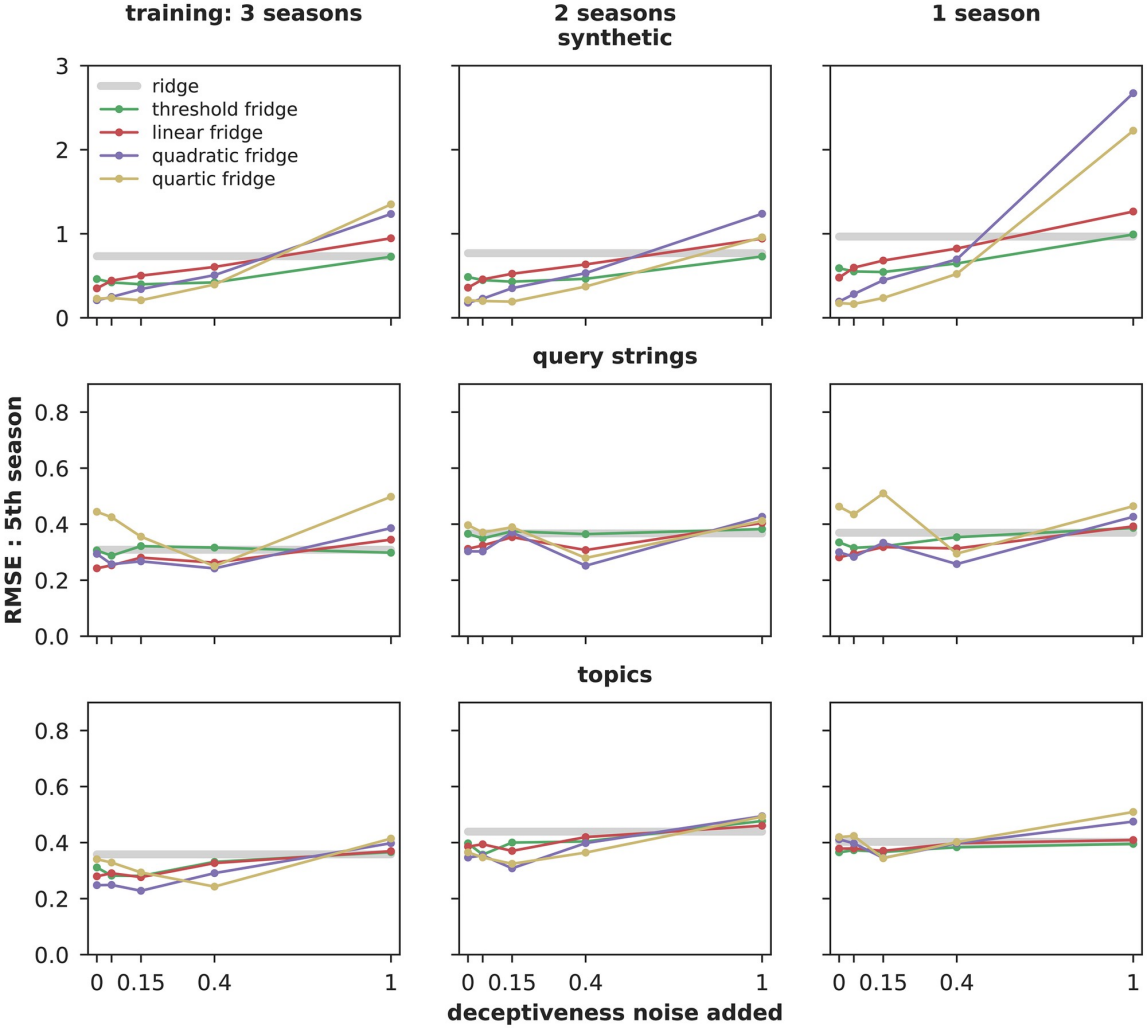
Gridge regression error compared to plain ridge, 5th season. Gridge algorithms that take into account deceptiveness *usually* have lower RMSE than plain ridge, which does not. Further, adding deceptiveness noise usually gives the appropriate trend: worse deceptiveness knowledge means worse predictions. *r*^2^ and RMSE on the 4th season show similar trends, while hit rate shows limited benefit from gridge; these figures are available in S1 Fig.

Fig 7 illustrates the effect on error (RMSE) of adding noise to deceptiveness information. In low-added-noise conditions (*γ* = 0 or 0.05), the gridge algorithms generally have lower error (median 0.35, range 0.15 − 0.65) than plain ridge (median 0.46, range 0.31 − 0.97), while in higher-added-noise conditions (*γ* = 0.40 or 1) their error is higher (median 0.41, range 0.24 − 2.7). That is, conditions with better knowledge of deceptiveness outperform the baseline, and performance declines as deceptiveness knowledge worsens, which is the expected trend. In addition, we find that quadratic and quartic algorithms tend to produce lower error than generalized ridge, threshold or linear fridge. Trends in *r*^2^ are similar; see S1 Fig. This supports our hypotheses that (a) incorporating knowledge of feature deceptiveness can improve estimates of disease incidence based on internet data and (b) semantic distance, as expressed in the Wikipedia article category tree, is an effective proxy for deceptiveness.

**Fig 8:**
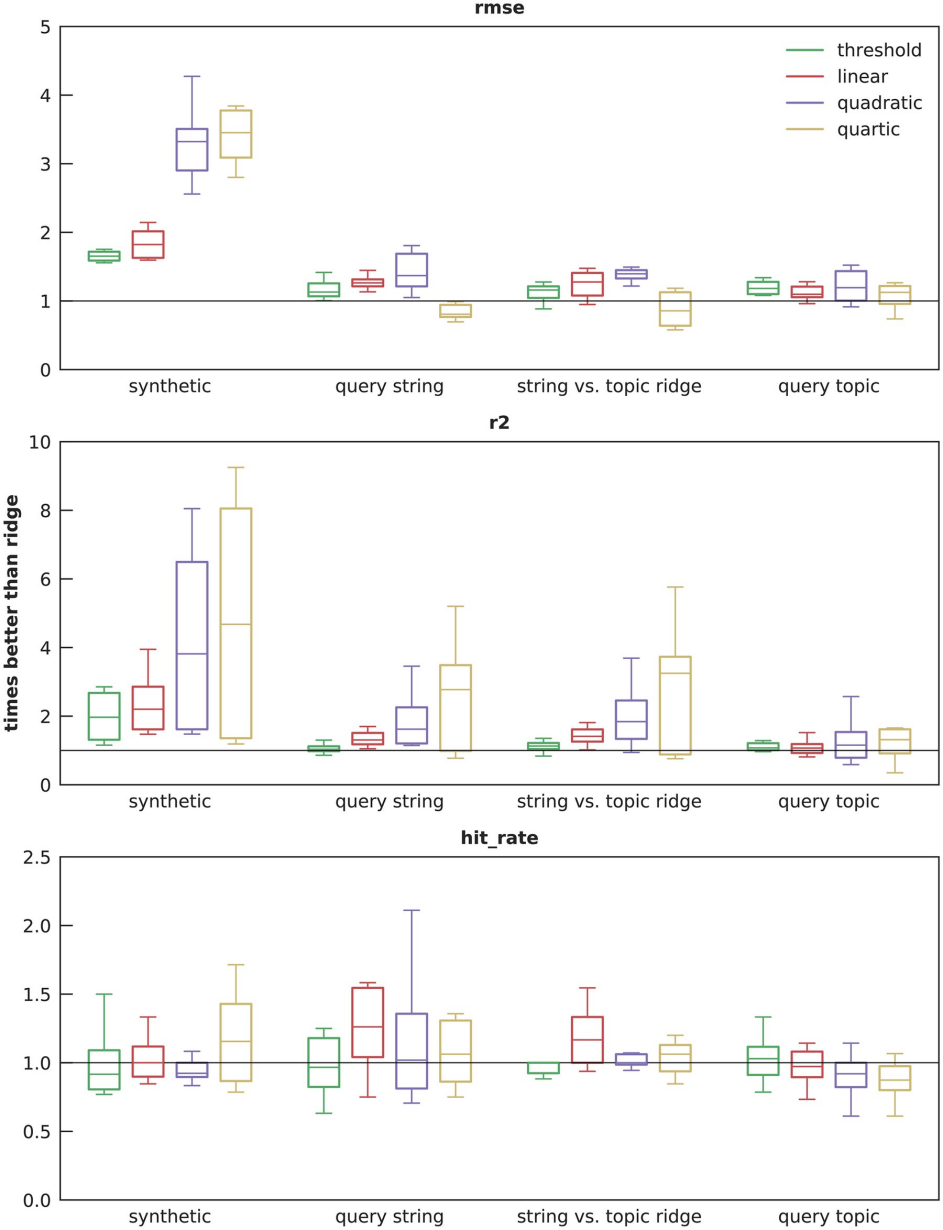
Improvement over ridge in zero- and low-noise conditions. This figure illustrates performance of the gridge algorithms divided by plain ridge in the same condition. The third group compares *query string* gridge models against *topic* ridge. Boxes plot the median with first and third quartiles, and the whiskers show the maximum and minimum. Each box-and-whiskers summarizes 12 data points. For the two error metrics, increasing the importance of deceptiveness through quadratic gridge yields increasing improvement over plain ridge, but the trend ends at quartic gridge. Notably, this is true even when comparing query string models (which are completely automatic) against topic ridge (which requires lots of manual attention), suggesting that deceptiveness information can be used to replace expensive human judgement. Hit rate, however, seems to gain limited benefit from deceptiveness in this experiment.

Fig 8 summarizes the improvement of the four gridge algorithms over plain ridge for all three metrics in the zero- and low-noise conditions (*γ* = 0 and 0.05). Over all feature types and metrics, the gridge algorithms perform a median of 1.4 times better (range 0.7 − 3.8). The best improvement is seen in synthetic features (median 2.1, range 0.8 − 6.0), while a more modest but still positive improvement is seen in topic features (median 1.06, median 0.6 − 2.3). These results suggest that adding low-noise knowledge of deceptiveness to ridge regression improves error. It also appears that the benefits of gridge level off between quadratic (deceptiveness squared) and quartic (deceptiveness raised to the fourth power): while quartic sometimes beats quadratic ridge, it frequently is worse than plain ridge.

We speculate that the lack of observed benefit of gridge on hit rate is due to one or both of two reasons. First, plain ridge may be sufficiently good on this metric that it has already reached diminishing returns; recall that in Fig 6 all five algorithms captured the overal trend of ILI well. Second, ILI is noisy, with lots of ups and downs from week to week regardless of the medium-term trend. This randomness may limit the ability of hit rate to assess performance on a weekly time scale without overfitting. That is, we believe that gridge’s failure to improve over plain ridge on hit rate is unlikely to represent a concerning flaw in the algorithm.

## Discussion

Our previous work introduced *deceptiveness*, which quantifies the risk that a disease estimation model’s error will increase in the future because it uses features that are coincidentally, rather than informatively, correlated with the quantity of interest [3]. This work tests the hypothesis that incorporating deceptiveness knowledge into a disease nowcasting algorithm reduces error; to our knowledge, it is the first work to quantitatively assess this question. To do so, we used simulated features with known deceptiveness as well as two types of real web search features with deceptiveness estimated using semi- and fully-automated algorithms.

### Findings

Our experiment yielded three main findings:

1. Deceptiveness information does help our linear regression nowcasting algorithms, and it helps more when it is more accurate.
2. A readily available, crowdsourced semantic relatedness measure, Wikipedia category distance, is a useful proxy for deceptiveness.
3. Deceptiveness information helps automatically generated features perform the same or better than similar, semi-automated features that require human curation.

The effects we measured are stronger for the synthetic features than the real ones. We speculate that this is for two reasons. First, the web search feature types are skewed towards low deceptiveness, because they are based on Wikipedia articles directly linked from “Influenza”, while the synthetic features lack this skew. Second, the synthetic features can have zero-noise deceptiveness information, while the real features cannot, because they use Wikipedia category distance as a less-accurate proxy. If verified, the second would further support the hypothesis that more accurate deceptiveness information improves nowcasts.

The third finding is interesting because it is relevant to a long-standing tension regarding how much human intervention is required for accurate measurements of the real world using internet data: more automated algorithms are much cheaper, but they risk oversimplifying the complexity of human experience. For example, our query strings were automatically generated from Wikipedia article titles, which are written for technical accuracy rather than salience for search queries entered by laypeople. To select features for disease estimation, one could use a fully-automated approach (e.g., our query strings), a semi-automated approach (e.g., our topics, which required a manual mapping step), or a fully-manual approach (e.g., by expert elicitation of search queries or topics, which we did not test).

One might expect that a trade-off would be present here: more automatic is cheaper, but more manual is more accurate. However, this was not the case in our results. The third box plot group in Fig 8 compares the gridge models using query string features to a baseline of plain ridge on *topic* features. Query strings perform favorably regardless of whether the baseline is plain ridge on query strings or topics, and sometimes the improvement is greater than gridge using topic features. This suggests that there is not really a trade-off, and fully automatic features might be among the most accurate.

There are real-world implications for these observations. Adding deceptiveness estimates can improve nowcasting of disease, and the amount of human attention needed to create good features may be minimal. In an operational context, disease models should be re-fit whenever new data become available; for example, in the U.S. for influenza, new ILI data are published weekly. The computational cost to fit our model is trivial, so this would not be a concern. Our related work investigates this question directly, finding that our observations hold in a real-world, real-time flu forecasting context [17].

### Limitations

All experiments are imperfect. Due to its finite scope, this work has many limitations. We believe that the most important ones are:

- Wikipedia is changing continuously. While we believe that these changes would not have a material effect on our results, we have not tested this.
- Wikipedia has non-semantic categories, such as the roughly 20,000 articles in category *Good article* that our algorithm that would assign distance 1 from each other. We have not yet encountered any other relevant non-semantic categories, and “Influenza” is not a *Good article*, so we believe this limitation does not affect the present results. However, any future work extending our algorithms should exclude these categories from the category distance computation.
- The mapping from Wikipedia articles to Google query strings and topics has not been optimized. While we have presented mapping algorithms that are reasonable both by inspection and supported by our current and prior [9] results, we have not compared these algorithms to alternatives.
- Linear regression algorithm metaparameters were not fully evaluated. For example, *ϵ* was assigned using our expert judgement rather than experimentally optimized.
- Other methods of feature generation may be better. This experiment was not designed to evaluate the full range of feature generation algorithms. In particular, direct elicitation of features such as query strings and topics should be evaluated.
- The deceptiveness metric itself is unrelated to semantic information; it is a measure of the risk of correlation changes. However, we use a deceptiveness proxy that is entirely driven by semantic relatedness. In the present work, this is a useful proxy that does appear to improve models, but it may not generalize to all other applications.

### Future work

This is an initial feasibility study using a fairly basic nowcasting model. At this point, the notion of deceptiveness for internet-based disease estimation is promising, but continued and broader positive results are needed to be confident in this hypothesis. In addition to addressing the limitations above, we have two groups of recommendations for future work.

First, multiple opportunities to improve nowcasting performance should be investigated. Additional deceptiveness-aware fitting algorithms such as generalized lasso [35] and generalized elastic net [36] should be tested. Category distance also has opportunities for improvement. For example, it can be made finer-grained by measuring path length through the Wikipedia category tree rather than counting the number of levels ascended: the distance between “Influenza” and “Infection” would become 3, taking into account that *Infectious diseases* was a direct category of the latter. Finally, alternate deceptiveness estimates need testing, for example category distance based on the medical literature. In addition to better nowcasting, utility needs to be demonstrated when deceptiveness-aware nowcasts augment best-in-class forecasting models, such as those doing well in the CDC’s flu forecasting challenge [14].

Second, we are optimistic that our algorithms will generalize well to different diseases and locations. This is because our best feature-generation algorithm is fully automated, making it straightforward to generalize by simply offering new input. For example, to generate features for dengue fever in Colombia, one could: start with the article “Dengue fever” in Spanish Wikipedia; write a set of Spanish stop phrases; use the Wikipedia API to collect links from that article and walk the category tree; pull appropriate search volume data from Google or elsewhere; and then proceed as described above. Future studies should evaluate generalizability to a variety of disease and location contexts.

### Conclusion

We present a study testing the value of deceptiveness information for nowcasting disease incidence using simulated and real internet features and generalized ridge regression. We found that incorporating such information does in fact help nowcasting; to our knowledge, ours is the first quantitative evaluation of this question.

Based on these results, we hypothesize that other internet-based disease estimation methods may also benefit from including feature deceptiveness estimates. We look forward to further research yielding deeper insight into the deceptiveness question.

## Supporting information

S1 Dataset Influenza-like-illness (ILI) data from the Centers for Disease Control and Prevention (CDC). These data are public domain. Our value-add is simply to clarify the dates and reformat the data.
Tab “ILINet_National” (unchanged download from the CDC):
**REGION TYPE**: Always “National”.**REGION**: Always “X”.**YEAR**: Year of row.**WEEK**: Week number of row.**AGE *n* − *m*** (6 columns): ILI case count in the specified age range.**ILITOTAL**: Total ILI case count (all ages).**NUM. OF PROVIDERS**: Number of reporting ILI providers.**TOTAL PATIENTS**: Total number of patients seen at reporting ILI providers.
Tab “data” (our reformatted version):
**Start date**: Sunday starting the week.**End date**: Saturday ending the week.**Year**: Year of row.**Week**: Week number of row.**provider_ct**: Number of reporting ILI providers.**national**: National ILI average, weighted by state population.
(XLS)

S2 Dataset Mapping of Wikipedia article URLs to Google search queries and topics. Row 1 gives totals of each column. Columns:
**article**: Wikipedia article URL.**raw query**: Google search query string derived from Wikipedia article URL.**topic**: Google search topic we think best matches the Wikipedia article.**topic code**: Hexadecimal code for Google search topic.**raw found**: 1 if query string found in the Google Health Trends API, 0 if not.**topic found**: 1 if topic found in GHT API, 0 if not.
(XLSX)

S3 Dataset Synthetic features used in our study. Column “Start date”: Sunday starting the week. Remaining columns: Value of synthetic feature *i* on that week.
(XLSX)

S4 Dataset List of raw queries used in Fig 1. (XLSX)

S1 Fig Error for metrics and seasons not shown in Fig 7. (PDF)
