# Supplementary material for: Estimating influenza incidence using search query deceptiveness and generalized ridge regression
Source: PLoS Comput Biol. 2019 Oct 1;15(10):e1007165. doi: 10.1371/journal.pcbi.1007165 (PMC6771994; doi:10.1371/journal.pcbi.1007165)
Supplement: S1 Fig — (PDF) [file pcbi.1007165.s005.pdf]

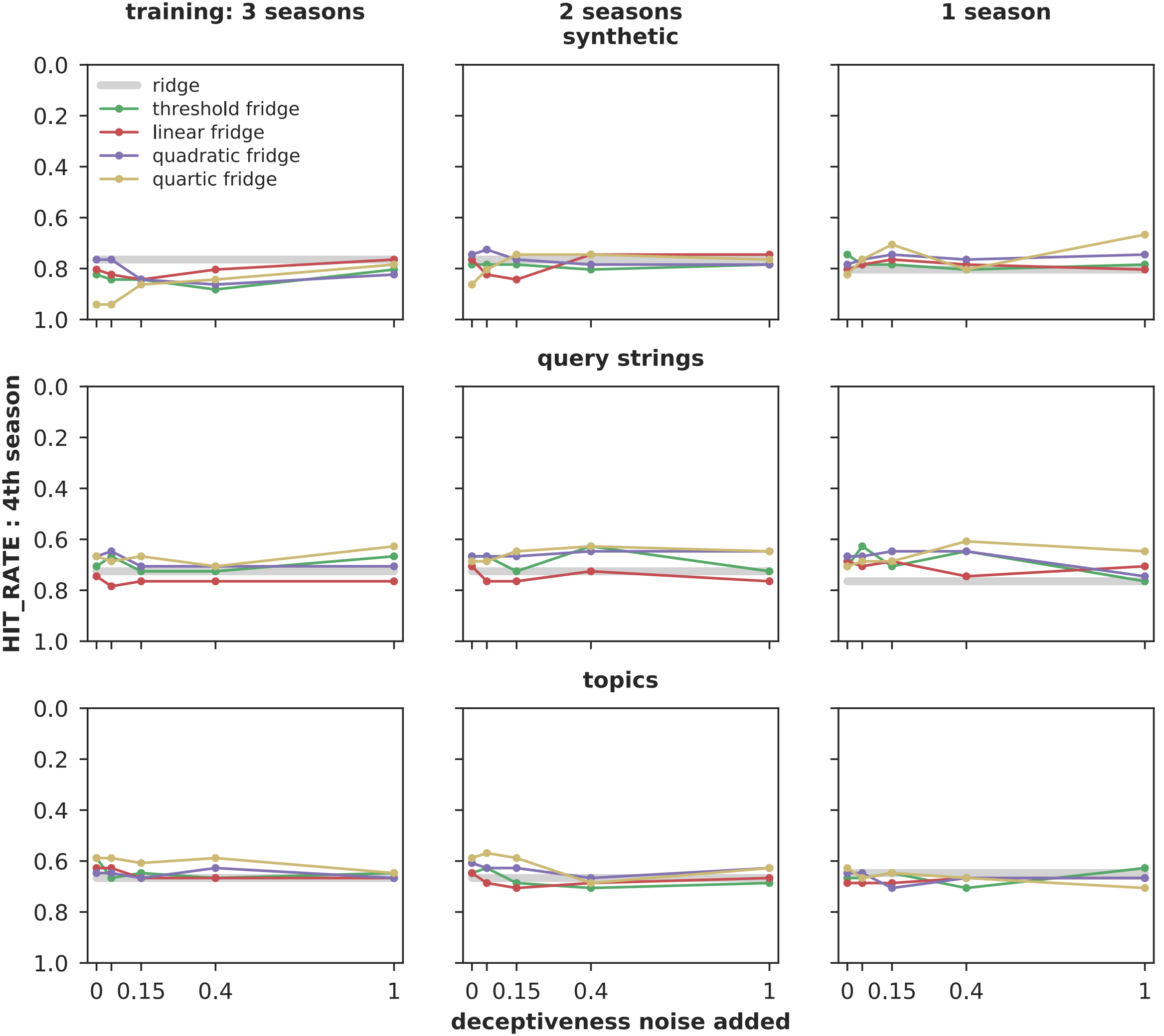
training: 3 seasons
2 seasons
synthetic
1 season
query strings
topics
ridge
threshold fridge
linear fridge
quadratic fridge
quartic fridge
HIT_RATE : 4th season
0.0
0.2
0.4
0.6
0.8
1.0
0
0.15
0.4
1
deceptiveness noise added

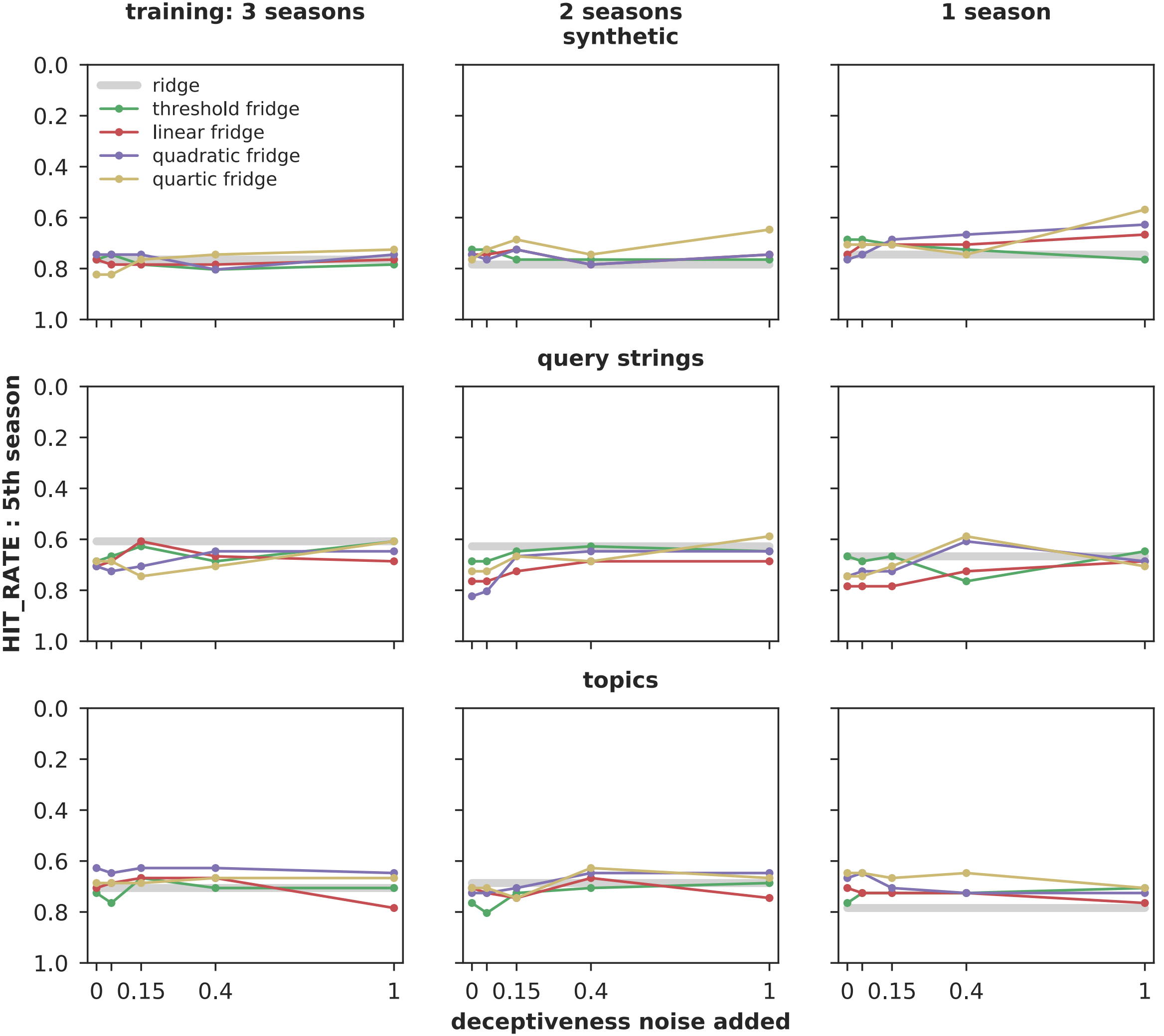
training: 3 seasons
2 seasons
synthetic
1 season
query strings
topics
ridge
threshold fridge
linear fridge
quadratic fridge
quartic fridge
HIT_RATE : 5th season
deceptiveness noise added
0.0
0.2
0.4
0.6
0.8
1.0
0
0.15
0.4
1

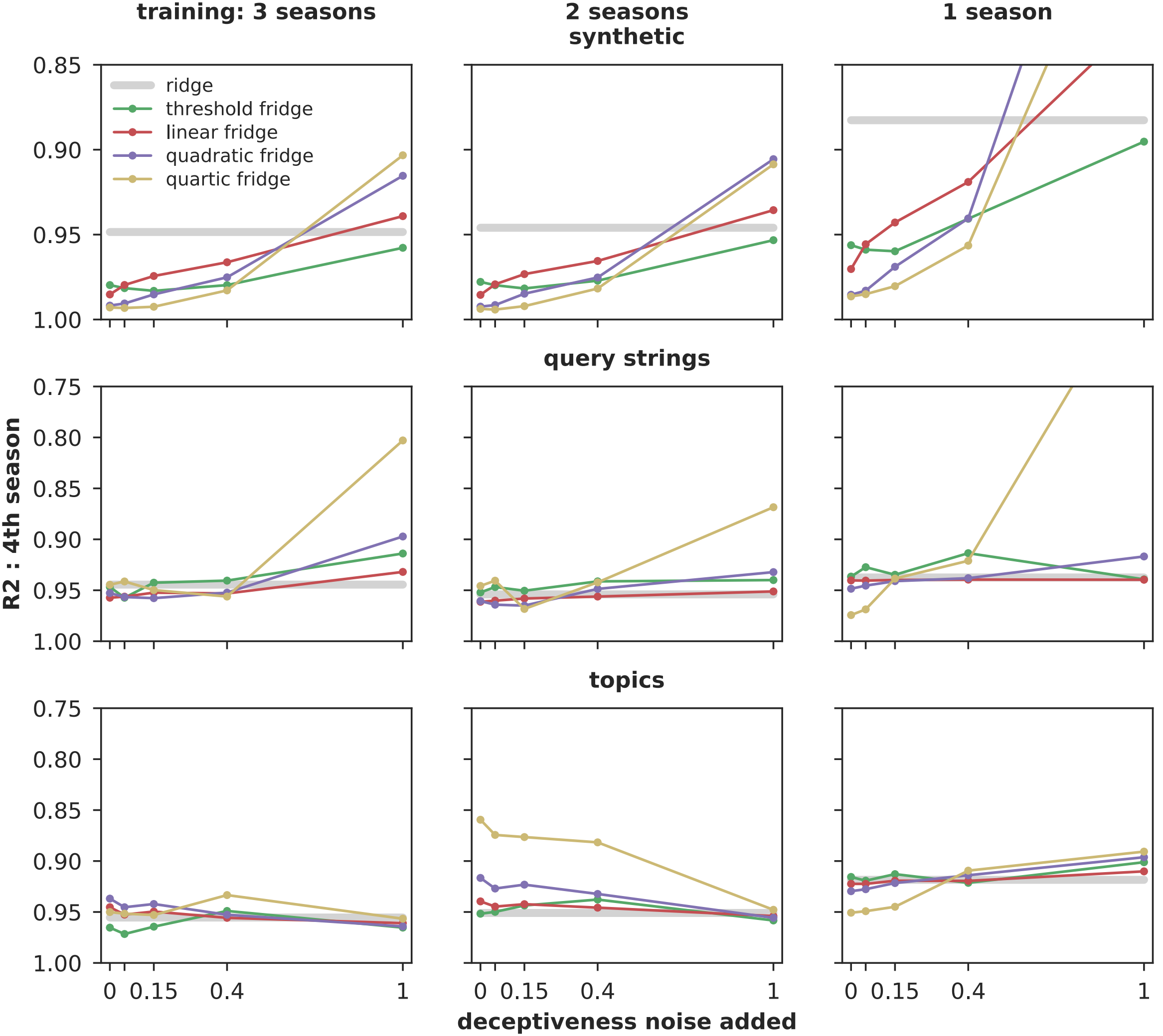

training: 3 seasons
2 seasons
synthetic
1 season
query strings
topics
ridge
threshold fridge
linear fridge
quadratic fridge
quartic fridge
R2 : 4th season
0.75
0.80
0.85
0.90
0.95
1.00
0
0.15
0.4
1
deceptiveness noise added

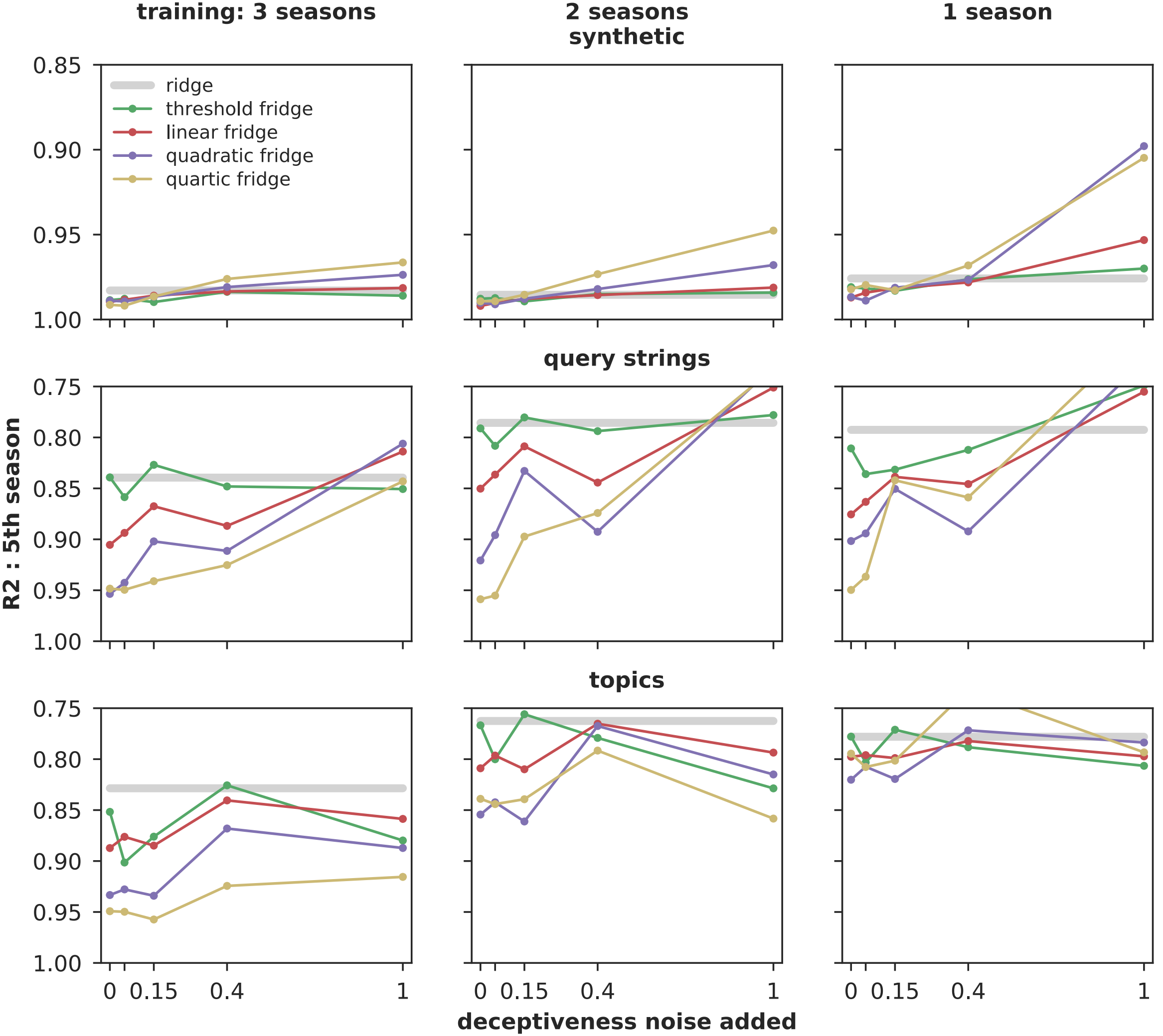
training: 3 seasons
2 seasons
synthetic
1 season
query strings
topics
ridge
threshold fridge
linear fridge
quadratic fridge
quartic fridge
0.85
0.90
0.95
1.00
0.75
0.80
R2 : 5th season
0
0.15
0.4
1
deceptiveness noise added

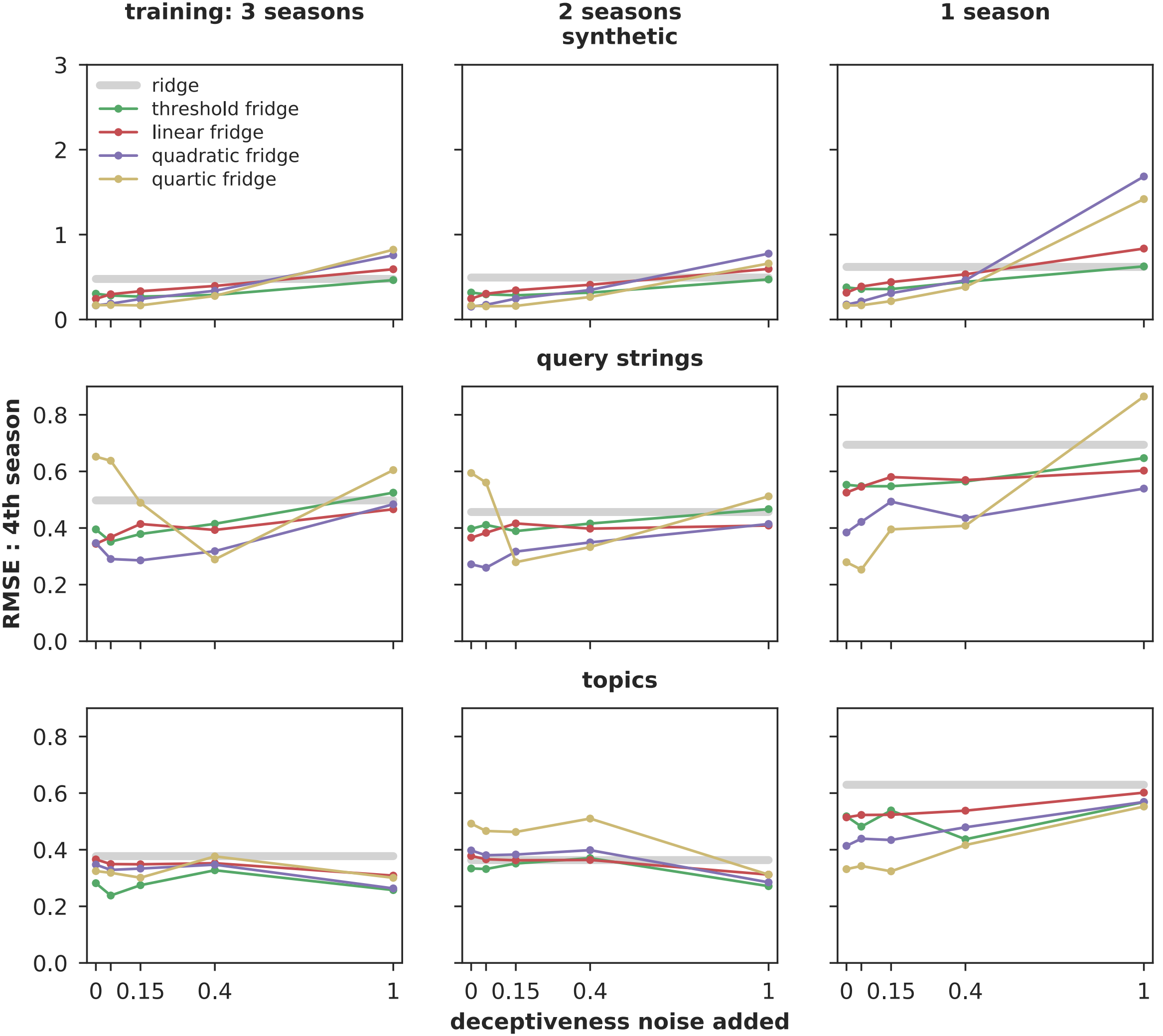

training: 3 seasons
2 seasons
synthetic
1 season
query strings
topics
ridge
threshold fridge
linear fridge
quadratic fridge
quartic fridge
RMSE : 4th season
deceptiveness noise added
0
0.15
0.4
1
0.0
0.2
0.4
0.6
0.8
2
3

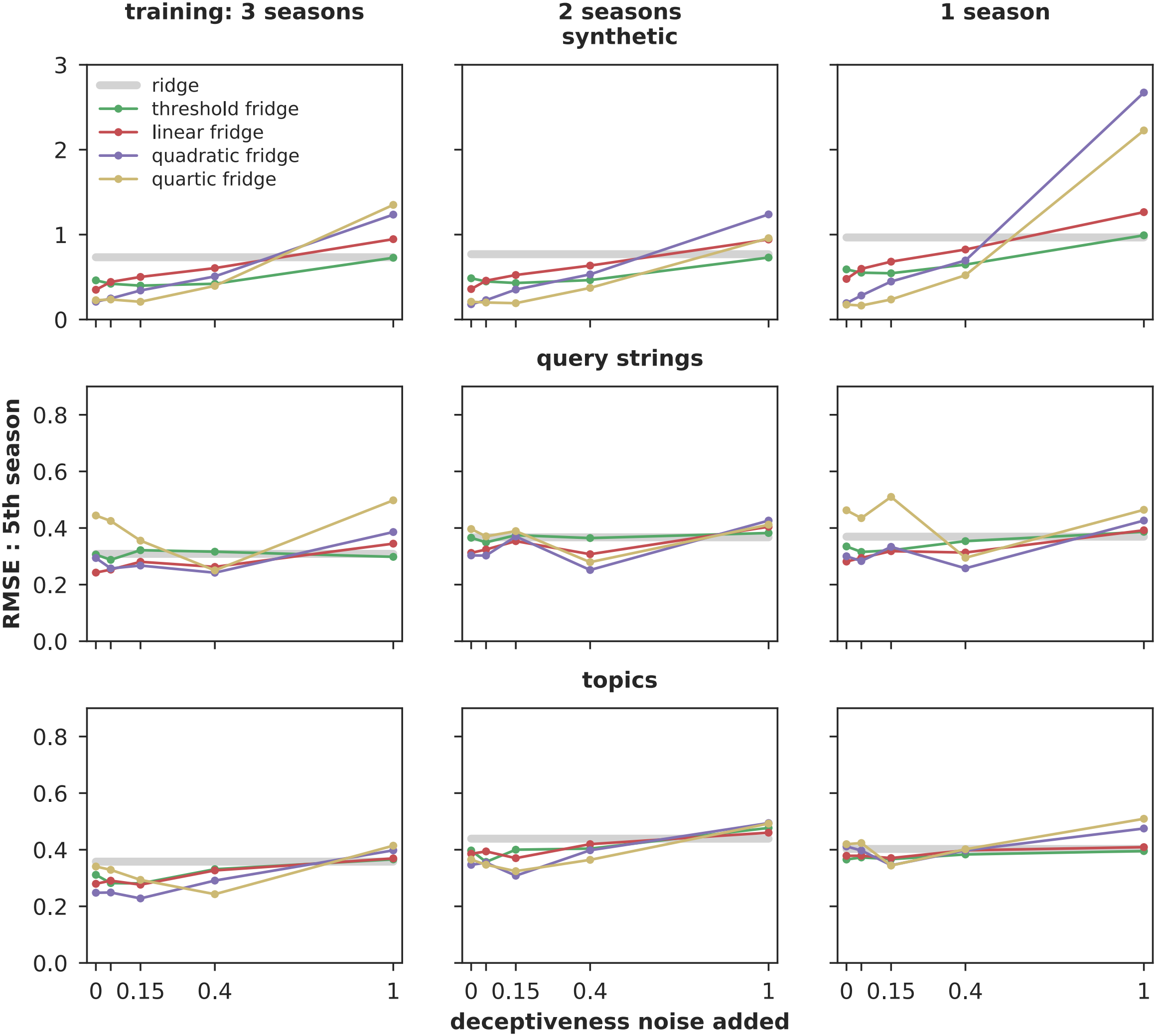
training: 3 seasons
2 seasons
synthetic
1 season
query strings
topics
ridge
threshold fridge
linear fridge
quadratic fridge
quartic fridge
RMSE : 5th season
deceptiveness noise added
0
0.15
0.4
1
0.0
0.2
0.4
0.6
0.8
2
3
